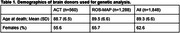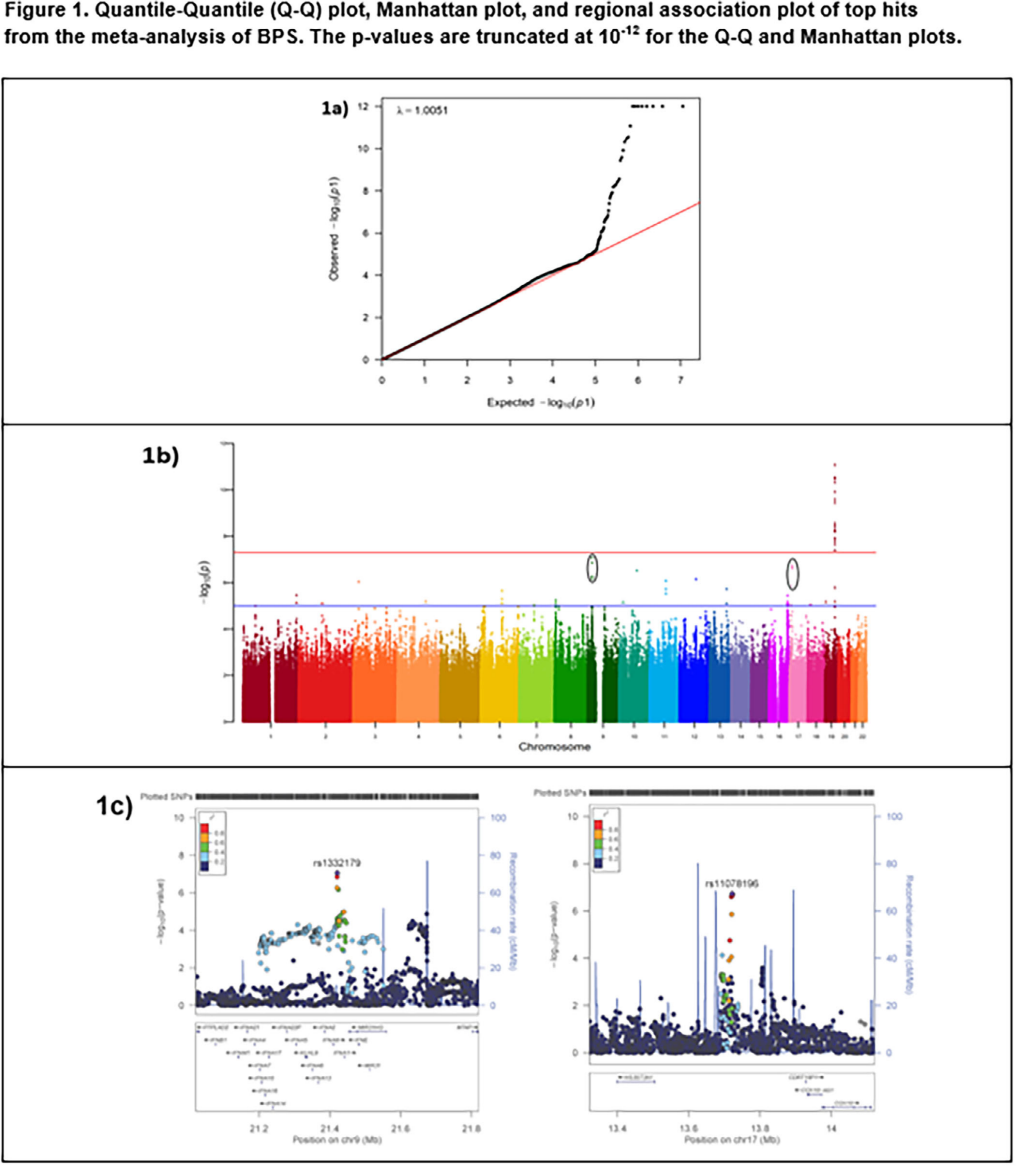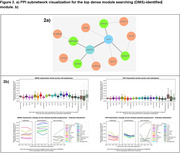# A systems‐biology approach to identify candidate genes associated with composite brain pathology scores in the Adult Changes of Thought (ACT) and the Religious Orders Study and Memory and Aging Project (ROSMAP) autopsy cohorts

**DOI:** 10.1002/alz.093129

**Published:** 2025-01-03

**Authors:** Aheli Dutta, Jai Broome, Sagnik Sinha, Laura E. Gibbons, Lincoln MP Shade, Yuriko Katsumata, Kyle J Travaglini, Mariano I Gabitto, Zijie Zhao, Qiongshi Lu, Julie A. Schneider, Caitlin S Latimer, C Dirk Keene, Paul K. Crane, David W. Fardo, Shubhabrata Mukherjee

**Affiliations:** ^1^ University of Washington, Seattle, WA USA; ^2^ University of Washington, School of Medicine, Seattle, WA USA; ^3^ College of Public Health, University of Kentucky, Lexington, KY USA; ^4^ Allen Institute for Brain Science, Seattle, WA USA; ^5^ University of Wisconsin School of Medicine and Public Health, Madison, WI USA; ^6^ Rush Alzheimer’s Disease Center, Rush University Medical Center, Chicago, IL USA

## Abstract

**Background:**

Previously, we developed a co‐calibrated and harmonized brain pathology score (BPS) across prospective cohort studies with research brain donation that incorporates multiple forms of postmortem neuropathology, using confirmatory factor analysis. We sought to identify genetic loci associated with BPS using a systems‐biology approach, combining data from participants in the Adult Changes in Thought (ACT), the Religious Orders Study, and Rush Memory and Aging Project (ROSMAP) autopsy cohorts.

**Method:**

We used PLINK in each cohort separately for genome‐wide association studies (GWAS) of BPS using HRC imputed data from European ancestry participants, adjusting for age at death, sex, and population substructure. We performed meta‐analysis using the adaptively weighted Fisher’s approach in METAL. We performed gene‐wide analysis using the meta‐analyzed results which we then integrated into the human protein‐protein interaction (PPI) network using a dense module searching (DMS) method to identify network hub genes for BPS. We interrogated the Seattle Alzheimer’s Disease Brain Cell Atlas (SEA‐AD) dataset on the middle temporal gyrus to determine which cell types both hub genes were expressed in and how they differed across donors with higher degrees of AD pathology (i.e. along AD pseudo‐progression).

**Result:**

The sample size consisted of 1,848 brain donors (**Table 1**). The quantile‐quantile plot and genomic inflation (λ = 1.005) for GWAS meta‐analyses showed no bias (**Figure 1a**), with the Manhattan plot in **Figure 1b**. Apart from significant SNPs around the *APOE* region, we identified two candidate loci a) (Chr 9: rs1332179; MAF = 0.1; P_meta = 8.7 × 10^‐8^) and b) (Chr 17: rs11078196; MAF = 0.34; P_meta = 1.9 × 10^‐7^). Regional association plots for these two loci are shown in **Figure 1c**. The PPI network analysis identified *VCP* and *IQCB1* as hub genes (**Figure 2a**). While both hub genes were expressed broadly across cell types, *IQCB1* was specifically higher with higher degrees of AD pathology in Microglia and *VCP* was lower with higher degrees of AD pathology in several neuronal populations (**Figure 2b**).

**Conclusion:**

We identified two potentially useful candidate loci associated with BPS using a systems‐biology approach. Further functional enrichment analysis is needed to determine whether these novel loci may identify targets for interventions to ameliorate AD.